# Impaired neural circuitry of hippocampus in *Pax2* nervous system‐specific knockout mice leads to restricted repetitive behaviors

**DOI:** 10.1111/cns.14482

**Published:** 2023-10-03

**Authors:** Ying Wang, Yizhuo Wang, Jiaming Tang, Rui Li, Yanan Jia, Hua Yang, Hongen Wei

**Affiliations:** ^1^ Department of Neurology, Shanxi Provincial People's Hospital The Fifth Clinical Medical College of Shanxi Medical University Taiyuan China; ^2^ Shanxi Key Laboratory of Brain Disease Control Shanxi Provincial People's Hospital Taiyuan China; ^3^ School of the Third Clinic Shanxi University of Chinese Medicine Taiyuan China

**Keywords:** hippocampus, IGFBP2, neural circuitry, *Pax2*, RRBs

## Abstract

**Introduction:**

Restricted repetitive behaviors (RRBs), which are associated with many different neurological and mental disorders, such as obsessive‐compulsive disorder (OCD) and autism, are patterns of behavior with little variation and little obvious function. Paired Box 2 (*Pax2*) is a transcription factor that is expressed in many systems, including the kidney and the central nervous system. The protein that is encoded by *Pax2* has been implicated in the development of the nervous system and neurodevelopmental disorders. In our previous study, *Pax2* heterozygous gene knockout mice (*Pax2* ^+/−^ mice) showed abnormally increased self‐grooming and impaired learning and memory abilities. However, it remains unclear which cell type is involved in this process. In this study, we deleted *Pax2* only in the nervous system to determine the regulatory mechanism of *Pax2* in RRBs.

**Methods:**

In this study, *Pax2* nervous system‐specific knockout mice (*Nestin‐Pax2* mice*)* aged 6–8 weeks and *Pax2* flox mice of the same age were recruited as the experimental group. Tamoxifen and vehicle were administered via intraperitoneal injection to induce *Pax2* knockout after gene identification. Western blotting was used to detect *Pax2* expression. After that, we assessed the general health of these two groups of mice. The self‐grooming test, marble burying test and T‐maze acquisition and reversal learning test were used to observe the lower‐order and higher‐order RRBs. The three‐chamber test, Y‐maze, and elevated plus‐maze were used to assess social ability, spatial memory ability, and anxiety. Neural circuitry tracing and transcriptome sequencing (RNA‐seq) were used to observe the abnormal neural circuitry, differentially expressed genes (DEGs) and signaling pathways affected by *Pax2* gene knockout in the nervous system and the putative molecular mechanism.

**Results:**

(1) The *Nestin‐Pax2* mouse model was successfully constructed, and the *Nestin‐Pax2* mice showed decreased expression of *Pax2*. (2) *Nestin‐Pax2* mice showed increased self‐grooming behavior and impaired T‐maze reversal behavior compared with *Pax2* flox mice. (3) An increased number of projection fibers can be found in the mPFC projecting to the CA1 and BLA, and a reduction in IGFBP2 can be found in the hippocampus of *Nestin‐Pax2* mice.

**Conclusion:**

The results demonstrated that loss of *Pax2* in the nervous system leads to restricted repetitive behaviors. The mechanism may be associated with impaired neural circuitry and a reduction in IGFBP2.

## Graphical abstract

The *Nestin‐Pax2* mice showed increased RRBs at both lower‐order and higher‐order. Furthermore, alterations in the mPFC→CA1/BLA neural circuitry and a reduction in IGFBP2 can be found in the hippocampus.

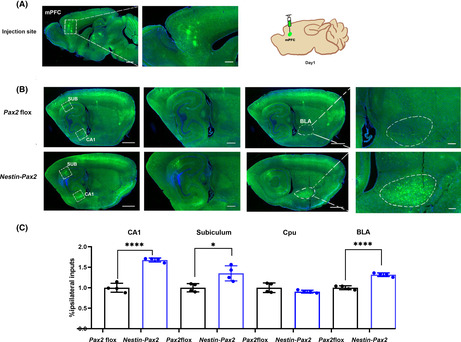

## 1 INTRODUCTION

Restricted repetitive behaviors (RRBs) are common behaviors across species and refer to a series of meaningless and repeated behaviors, including stereotyped body movements, narrow and restricted interests, repeated self‐injuries, repetitive motion of speech and cognitive inflexibility. Existing studies classified RRBs into lower‐order RRBs and higher‐order RRBs.^1^, ^2^, ^3^ Lower‐order RRBs refer to physical, repetitive behaviors such as stereotyped movements with or without objects and self‐injuries. While higher‐order RRBs tend to persist in sameness or resistance to change, the inflexibility and rigidity of perception are even more important. RRBs are strongly associated with many neurodevelopmental disorders, such as autism spectrum disorder (ASD), Rett syndrome, fragile X syndrome (FXS), and Prader–Willi syndrome. Meanwhile, RRBs also share the phenotype of other central nervous system disorders, including obsessive‐compulsive disorder (OCD), Tourette's syndrome, schizophrenia, and Alzheimer's disease (AD).^4^, ^5^ Current studies strongly suggest that both environmental factors and genetic risk factors play a crucial role in the pathogenesis of RRBs.^1^, ^3^, ^6^ Many mouse models of RRBs also show alterations in synaptic function and structure, excitation–inhibition balance and neuroinflammation, all of which are biological processes involved in the occurrence of RRBs.^7^ However, the exact pathophysiology of RRBs remains unclear.

Recent research suggests that transcription factors play an increasingly important role in the process of neurodevelopment. Paired Box 2 (*Pax2*) is a member of the paired‐box transcription factor family that is expressed in many tissues, including the kidney, optic nerve, ear, pancreas, and central nervous system,^8^ and is critical for the development of the kidney and central nervous system in humans and mice.^9^ *Pax2* is expressed in distinct regions of the developing central nervous system, particularly in the developing forebrain, midbrain, and hindbrain during the early stages of embryogenesis and later in the midbrain–hindbrain boundary, diencephalon and cerebellum. Patients with *Pax2* mutations exhibit various neurodevelopmental disorders, such as ASD, epilepsy, intellectual disability, and developmental delay.^10^, ^11^ In a previous study, we investigated the behavioral phenotype and possible underlying mechanism in *Pax2* heterozygous gene knockout mice (*Pax2* ^+/−^ mice). The *Pax2* ^+/−^ mice showed increased self‐grooming behavior and impaired spatial and memory abilities, which were attributed to microglia‐mediated synaptic dysfunction.^12^, ^13^ Due to the limitations of heterozygous gene knockout, we generated *Pax2* nervous system‐specific knockout mice (*Nestin‐Pax2*) to reduce the impact of other systems on the research.

However, individual alterations in synaptic function or targeted neurotransmitter systems are not sufficient to elucidate the pathophysiology of RRBs generated by neurofibrillary tangles from different functional brain regions. The underlying neurobiological substrates and neural circuitry involved in RRBs remain largely unknown. The prefrontal cortex is extensively connected to other brain areas, such as the hippocampus, amygdala, and thalamus, and thus participates in and regulates many brain functions.^14^ Previous research on animal models of RRBs has been devoted to understanding the pathophysiology of RRBs by examining dysfunction of the cortico‐striato‐thalamo‐cortical (CSTC) neural circuitry.^15^ Recent neuroimaging evidence suggests that areas of the brain other than those related to the CSTC neural circuitry are involved in the development of RRBs. The hippocampus is a critical structure involved in spatial and nonspatial memory. The hippocampus can be functionally divided along its longitudinal axis into dorsal, middle, and ventral parts and along the transverse axis into CA1, CA3, and the dentate gyrus (DG). Studies have shown that the hippocampus is an important brain area for information transmission to the prefrontal cortex and basolateral amygdala (BLA).^16^ In rodents, the prefrontal nerve projects to the hippocampus and BLA, forming some nerve fiber connectivity that plays an important role in the generation and regulation of behavior.

To further investigate whether *Pax2*‐specific knockout in the nervous system affects the behavioral phenotype of mice and how *Pax2* affects information processing in the nervous system, we evaluated the behaviors of *Nestin‐Pax2* mice in various aspects. Neural circuitry tracing and transcriptome sequencing (RNA‐seq) were used to observe the abnormal neural circuitry, differentially expressed genes (DEGs), and signaling pathways affected by *Pax2* gene knockout in the nervous system and the putative molecular mechanism.

## 2 MATERIALS AND METHODS

### 2.1 Animals and housing

**FIGURE 1:**
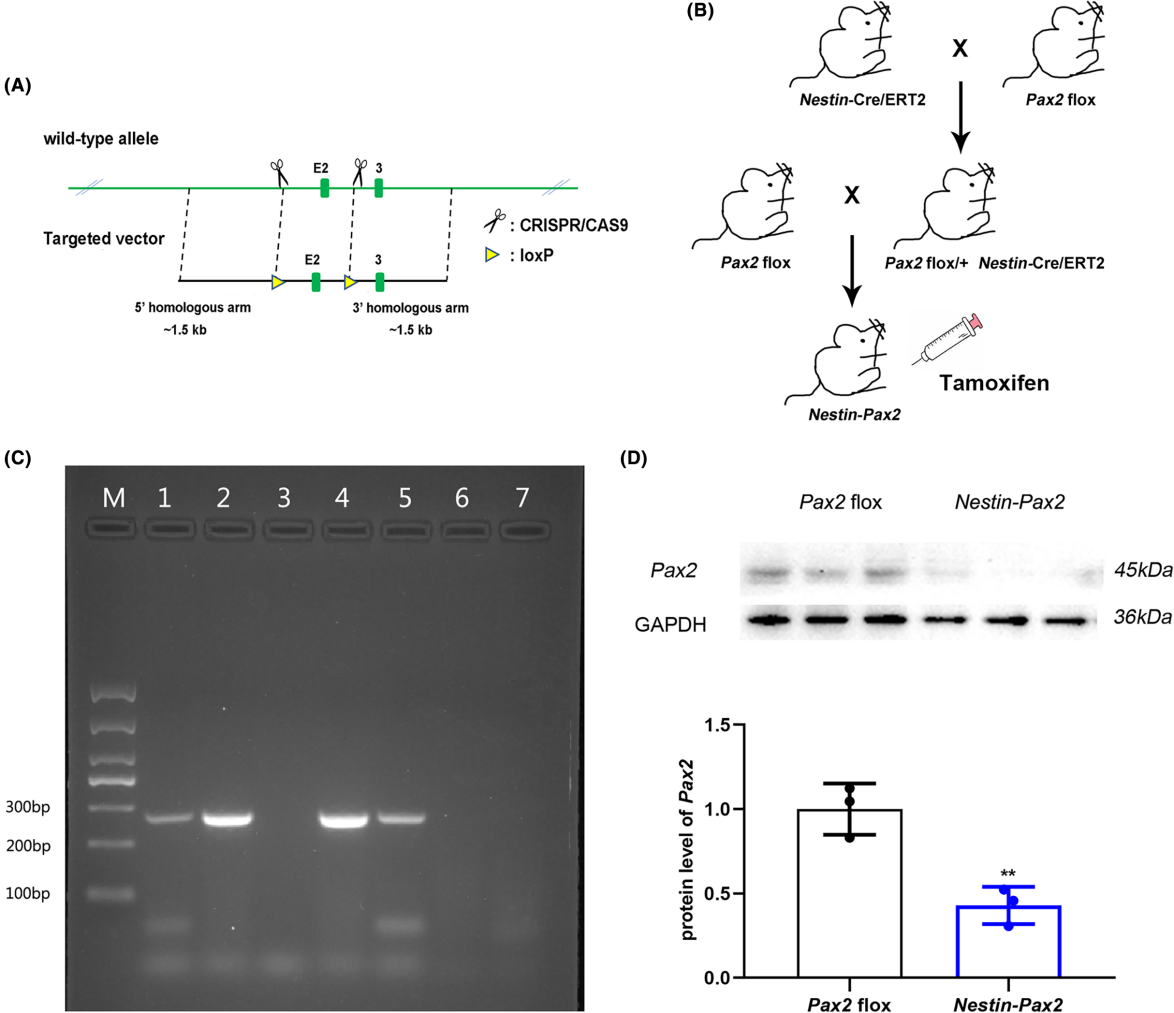
Construction of *Nestin‐Pax2* mice using the Cre‐loxp system. (A) Targeting strategy. (B) Generation of *Nesin‐Pax2* mice and *Pax2* flox mice. (C) PCR identification of *Nestin‐Pax2* mice. M: marker; 1, 2, 4 and 5: *Nestin‐Pax2*; 3, 6 and 7: *Pax2* flox. (D) Alternation of *Pax2* protein levels between *Nestin‐Pax2* mice and *Pax2* flox mice using Western blotting. Data are shown as the mean ± SEM. *p < 0.05, **p < 0.01.

Nestin‐CreER mice were purchased from Shanghai Model Organisms Center, Inc. *Pax2* ^flox/flox^ mice were generated by Bcgen (Beijing Biocytogen Co., Ltd). For the generation of *Pax2* ^flox/flox^ mice, the targeting vector included the 5′ homology arm (1.4 kb), the 5′ loxP site, the *Pax2* exon 2, the 3′ loxP site, and the 3′ homology arm (1.4 kb). Clones were analyzed by PCR and Southern blotting (LR probe for the 5′ end and 3′ probe for the 3′ end). In the wild‐type *Pax2* locus, the LR probe hybridized to a 9.6 kb fragment and to a 6.0 kb fragment in the *Pax2* loxp allele. The 3′ probe hybridized to a 14.6 kb fragment in the wild type or to a 6.0 kb fragment in the *Pax2* loxp allele (Figure 1A). We first generated Nestin‐CreER: *Pax2* ^flox/+^ mice by crossing Nestin‐CreER with *Pax2* ^flox/flox^ mice. Nestin‐CreER: *Pax2* ^flox/+^ mice were further crossed with *Pax2* ^flox/flox^ (*Pax2* flox) mice to obtain Nestin‐CreER: *Pax2* ^flox/flox^ (*Nestin Pax2*) mice. Littermates with other genotypes (i.e., *Pax2* flox) were used as controls. The mice were bred at the Laboratory Animal Center of Shanxi Provincial People's Hospital. The experimental mice were weaned at 21 ± 1 days of age and then grouped according to sex and strain in standard mouse cages with 2–4 mice per cage. Standard mouse chow and tap water were provided freely. The colony room was kept on a 12:12 light/dark cycle. All experiments were approved by the Shanxi Provincial People's Hospital Laboratory Animal Center and were in accordance with the ARRIVE guidelines for the care and use of laboratory animals.

### 2.2 Mouse genotyping

Mutant mice were genotyped by PCR using tail DNA with primers (F: AGCGATGGATTTCCGTCTCTGG; R: AGCTTGCATGATCTCCGGTATTGAA) and DNA sequencing.

### 2.3 Tamoxifen induction

To activate Cre‐mediated recombination, tamoxifen treatment was performed at the age of 4–6 weeks. Tamoxifen (Sigma–Aldrich, T5648) was dissolved in corn oil at 10 mg/mL. In addition, 1.0 mL of corn oil containing 5% ethanol was prepared for vehicle injection. Each mouse was injected intraperitoneally with 100 μL of tamoxifen stock solution (equivalent to 1 mg tamoxifen) or vehicle once every 24 h for five consecutive days.

### 2.4 Stereotaxic injection

All operations were performed under aseptic conditions using an animal stereotactic instrument (RWD Life Science). For viral tracing experiments, HSV viruses carrying the gene for enhanced green fluorescent protein (HSV‐EGFP) were obtained from Brain VTA, Wu Han, China. The viruses were injected using the same system described above in a biosafety level 2 (BL2) house. Mice were anesthetized with 1% pentobarbital sodium (1 g/100 mL). The HSV virus was injected into the left mPFC (AP: 1.78 mm, ML: 0.25 mm, DV: −2.75 mm). Virus volumes were 200 nL for the projection experiments. After the injection, the needle was left in place for 10 min to ensure that the virus spread to the target site before being slowly withdrawn. The mice were kept on an electric blanket until they fully recovered from anesthesia. The mice were housed in a BL2 house after surgery. Three days after HSV virus injection, the animals were used for experiments.

### 2.5 Histology and fluorescence imaging

Animals were perfused intracardially with PBS followed by 4% paraformaldehyde (PFA) in phosphate‐buffered saline. Brains were removed from the skulls and stored in 4% PFA at 4°C for 24 h and cryoprotected in 30% phosphate‐buffered sucrose at 4°C for 3 days. Brains were sliced sagittally (40 μm thick) and then immunostained with 4′, 6‐diamidino‐2‐phenylindole (DAPI). Fully stained samples were imaged using an automated section scanning system (Olympus vs120) with a 2× objective.

### 2.6 Behavior tests

Behavioral testing was conducted in a dedicated behavioral testing room during the standard light phase. Mice were placed at least 1 h before the start of the behavioral test in a holding room located in the hallway of the testing area. Tests were performed between 0900 and 1700 h. All animals in the behavioral tests were 6–8 weeks of age. In view of the age of the mice and the effect between behavioral tests, we divided the mice into two groups. The testing order of the first group was as follows: (1) general health check and neurological screening, (2) self‐grooming test, (3) social approach, (4) elevated‐plus maze test, (5) Y‐maze, and (6) marble burying test. The testing order of another group of mice was (1) Spray‐induced self‐grooming test, (2) T‐maze acquisition and reversal learning test.

#### 2.6.1 General health check and neurological screening

General health check and neurological screening include assessing body weight, fur and whisker condition, limb and body tone, sensory function and neurological reflexes. The fur condition was scored on a scale of 1–3, with 3 being normal healthy fur. Body tone and limb tone were scored on a scale of 1–3, with 3 = stiff, 2 = normal, and 1 = flaccid. Sensory function tests included the visual placing test and Preyer reflex test. In the visual placing test, the mouse is held by its tail at a height of approximately 15 cm above a table. The mouse extends its front paws for a ‘soft landing’ as it is gradually lowered onto the table. Blind mice did not see the approaching surface and did not raise their paws until their whiskers or nose touched the table. Extending forepaws was recorded as a yes or no response by the experimenter. The Preyer reflex is a flinching response to the sound of a loud handclap. The reflex was considered positive if the animal was seen to move quickly, using the whole body. The neurological screening tests were designed to detect any gross abnormalities in body function. The ear twitch reflex occurred when the auricle was touched from behind with a cotton swab, resulting in an immediate movement of the ear. The eye‐blink reflex occurred when a cotton swab was drawn once near the eye, resulting in a blink. The postural reflex was test by placing the mouse in an empty cage and shaking the cage to elicit extension of all 4 legs to maintain an upright, balanced position. The righting reflex was tested by turning the mouse onto its back and eliciting an immediate turning response to restore upright posture on all 4 legs. The whisker‐touch reflex was tested by gently touching the whiskers of a freely moving mouse; normal mice would stop moving their whiskers and turn their head toward the facet where the whiskers were touched.

#### 2.6.2 Self‐grooming test

Mice were assessed for spontaneous repetitive self‐grooming. In brief, each mouse was individually placed in a clean, empty mouse cage with no bedding. Each mouse was allowed to acclimate for 10 min in an empty cage. The cumulative time spent grooming all body regions was then scored for 10 min. The pattern of grooming in rodents usually proceeds in a cephalocaudal direction and involves several distinct phases: phase 1, paw and nose grooming; phase 2, face grooming; phase 3, head grooming; and phase 4, body grooming. Based on the above phases, a grooming bout was considered “interrupted” if at least one interruption was recorded within its transitions. Interruptions longer than 6 s were considered separate grooming phases. The cumulative time spent grooming and the number of grooming bouts were recorded with a stopwatch by the investigator sitting approximately 2 m from the test cage. Key ethological measures of grooming patterns included the cumulative time spent grooming, the number of grooming bouts within 10 min, the percentage of interrupted grooming bouts and the average bout length (time spent grooming/the number of grooming bouts).

#### 2.6.3 Social approach

Social approach behavior was tested in a socialization apparatus consisting of three chambers (20 × 40 × 22 cm). These three chambers were separated by two sliding doors (5 × 8 cm). These doors could be closed to confine the animal. Prior to the sociability test phases, this test had two habituation phases (center and all three chambers). The test is a comparison of preference between a social stimulus and an inanimate object. In a single 30‐min session, divided into three phases, the social approach behavior was tested in the apparatus. The subject mouse was habituated to the apparatus for 10 min in the central chamber (Phase 1) and then given access to all three empty chambers for an additional 10 min (Phase 2). The subject was then confined to the center chamber, while the novel object (an inverted wire cup) was placed in one of the side chambers, and the strange mouse, in an identical inverted wire cup, was placed in the opposite side chamber. C57BL/6 mice of the same sex were used as strange mice. The location (left or right) of the novelty object and strange mouse alternated across subjects. The chamber doors were opened simultaneously, and the subject had access to all three chambers for 10 min (Phase 3). Time spent in each of the three chambers and time spent sniffing were automatically scored by video tracking with the SuperMaze system (Shanghai Xinruan Information Technology Co. Ltd.). For each subject, the time spent sniffing the wire cage containing the foreign mouse and the time spent sniffing the empty wire cage were recorded. The sniffing time directed at each wire cage was scored cumulatively over each 10‐min test session as the total time in seconds. The foreign mice were habituated to the test chamber and confined to the wire cage for 30‐min sessions on three consecutive days. This ensured that all social approaches were initiated by the test mouse. Both end chambers were illuminated at 26–27 lx with 2 desk lamps angled away from the maze.

#### 2.6.4 The elevated‐plus maze test

The elevated‐plus maze has the shape of a +, and the apparatus consists of two open arms (35 × 5 cm) and two closed arms (30 × 5 × 15 cm) extending from the same central platform (5 × 5 cm). The maze was raised to a height of 60 cm above the ground. The test was conducted in a quiet room with dim lighting. Each mouse was individually placed in the center of the maze with its head facing toward one of the open arms, and its behavior was monitored for a duration of 5 min using SuperMaze software. The time spent in the open and closed arms was recorded. After each trial, the maze was wiped with 75% of ethanol and allowed to dry completely to eliminate olfactory cues before the next trial could begin.

#### 2.6.5 Y‐maze

The Y‐maze test is used to measure immediate spatial working memory, which is a form of temporary memory. The Y‐maze is composed of three arms at equal angles (40 cm in length × 10 cm in width × 12 cm in height). The mice were placed at the end of one of the arms and were allowed to freely explore the maze for a period of 5 min. The order in which the arms were entered was recorded visually, and the proportion alternated was calculated once. In addition to repetition, spontaneous alternation was described as entering all three arms consecutively. The proportion of alternation was calculated once as the ratio of the actual to the possible alternations (defined as the total number of arm entries minus two) multiplied by 100 according to the following equation: Alternation % = [number of alternations/(total number of arm entries − 2)] × 100. As an indicator of locomotor activity, the number of arm entries was also used. After each mouse, the maze was cleaned with 75% of ethanol and dried completely to eliminate the olfactory cues.

#### 2.6.6 Marble burying test

We used standard polycarbonate mouse cages (40 × 25 × 15 cm) fitted with filter top covers and 5 cm deep bedding material. Twenty glass toy marbles (approximately 15 mm in diameter) were gently placed on the surface of the bedding in five rows of four marbles each. The mice were then placed in a corner of the cage and recorded for 30 min. After each trial, the marbles were cleaned with 75% of ethanol to prevent olfactory cues. A marble was scored as buried when two‐thirds of its surface was covered by bedding. The parameter scored was the number of buried marbles.

#### 2.6.7 Spray‐induced self‐grooming test

After 10 min of habituation, pure water was sprayed 2–3 times on the head of the mouse at a distance of 20–35 cm, and the subsequent grooming behavior of the mouse was recorded within 10 min for further analysis. The above test indicators should also be analyzed.

#### 2.6.8 T‐maze acquisition and reversal learning test

The T‐maze was constructed of light blue organic board, with a 71 cm stem section and two 46 cm arm sections. Each section was 10 cm wide with 10 cm high walls. Prior to the test, the mice were deprived of food and reduced to approximately 85% of their free‐feeding body weight. The mice were first habituated to the T‐maze and trained to obtain food from the ends of the arms for 5 days, with one habituation trial per day. Ten training trials per day were then started. For each mouse, one arm with food reward was designated as the correct arm. At the beginning of each test session, the mouse was placed at the end of the T‐maze stem. The door of the stem section was then opened, and the mouse was given 60 s to make a choice between entering either arm. If the mouse made the correct choice, it was given time to consume the reward and then returned to the stem arm for the next trial. If it made the wrong choice, it stayed in the arm for 60 s and then was guided back into the stem for the next trial. On each subsequent trial, the reward was always placed in the same arm. The latency between trials was 30 s. The criterion for task acquisition was an average of 80% correct responses over 3 days of testing. When the mice reached the criterion, they were further tested using a reversal procedure. In the reversal procedure, the reward is placed in the opposite arm, and the above steps are repeated for 5 days. The number of errors in each trial was recorded.

### 2.7 Western blotting

The animals were sacrificed by decapitation, and the cerebral cortex was rapidly dissected. RIPA buffer was then used to homogenize these brain sections. Lysate concentrations were then normalized and denatured in loading buffer at 95°C and stored at −20°C until use, after which a Bradford assay was performed to calculate protein yield. One hundred micrograms of lysate were electrophoresed on 10% SDS–PAGE gels and separated. Gels were transferred to PVDF membranes and incubated in 5% of nonfat milk solution for 2 h at room temperature. Blots were incubated with primary antibody overnight at 4°C, washed four times for 15 min in PBS containing 0.1% Tween 20, followed by incubation with horseradish peroxidase‐conjugated goat anti‐rabbit (1:20000, EarthOx, ID: E030120‐01) for 1 h. Blots were visualized with chemiluminescent substrate (Millipore) after washing four times for 15 min. The following antibodies were used: rabbit anti‐Pax2 (1:200, Boster Biological Technology, ID: PB9734) and rabbit anti‐GAPDH (1:5000, Bioworld Technology, ID: AP0060). Quantification of band intensity was performed using ImageJ (NIH, Bethesda, Maryland) and presented relative to the internal control (GAPDH).

### 2.8 RNA isolation, cDNA library preparation, and sequencing

Total RNA was extracted with TRIzol (Invitrogen) and evaluated with an Agilent 2100 BioAnalyzer (Agilent Technologies) and Qubit Fluorometer (Invitrogen). Subsequent experiments used total RNA samples that met the following requirements: RNA integrity number (RIN) > 7.0 and a 28S:18S ratio > 1.8. RNA‐seq libraries were generated and sequenced by CapitalBio Technology. The triplicate samples of all assays were constructed as an independent library, and the following sequencing and analysis were performed. The NEB Next Ultra RNA Library Prep Kit for Illumina (NEB) was used to create the libraries for sequencing. The NEB Next Poly(A) mRNA Magnetic Isolation Module (NEB) kit was used to enrich for the poly(A)‐tailed mRNA molecules from 1 g of total RNA. The mRNA was fragmented into ~200 base pair pieces. First strand cDNA was synthesized from mRNA fragments with reverse transcriptase and hexamer random primers, and then second strand cDNA was synthesized using DNA polymerase I and RNaseH. The end of the cDNA fragment underwent an end repair procedure involving the addition of a single A base followed by ligation of the adapters. Products were purified by polymerase chain reaction (PCR) and enriched to amplify library DNA. The final libraries were quantified using the KAPA Library Quantification Kit (KAPA Biosystems, South Africa) and an Agilent 2100 Bioanalyzer. After quantitative reverse transcription polymerase chain reaction (RT–qPCR) validation, libraries were subjected to paired‐end sequencing with a pair‐end read length of 150 base pairs on an Illumina NovaSeq sequencer (Illumina).

### 2.9 RNA‐seq: data analysis

The genome of the human genome version of hg38 was used as a reference. Sequencing quality was assessed using FastQC (v0.11.5), and then low‐quality data were filtered using NGSQC (v2.3.3). The clean reads were then aligned to the reference genome using HISAT2 (v2.1.0) with default parameters. The processed reads from each sample were matched against the reference genome using HISAT2. The gene expression analyses were performed with StringTie (v1.3.3b). DESeq (v1.28.0) was used to analyze the DEGs between samples. Thousands of independent statistical hypothesis tests were performed separately on DEGs. Then, a *p* value corrected by the FDR method was obtained. The corrected *p* value (*q* value) was calculated by correcting using the BH method. *p* values or *q* values were used to perform a significance analysis. Parameters for classifying significant DEGs are 2‐fold differences (|log2FC| 1, FC: the fold change of expression) in transcript abundance and *p* < 0.05. The DEGs were annotated using information from the ENSEMBL, NCBI, UniProt, GO and KEGG databases.

### 2.10 Gene ontology and pathway enrichment analysis

Candidate differentially expressed genes (DEGs) were subjected to functional and pathway enrichment analysis using multiple online databases, among which DAVID is a repository of gene annotation, visualization, and integrated discovery capabilities, thus providing biological meaning to gene sequences. Enrichment analysis of GO and KEGG terms was conducted using the goana and kegga functions of the limma R package.

### 2.11 Protein–protein interaction network analysis

Protein–protein interaction (PPI) network analysis was conducted by first employing the STRING database (available online at http://string‐db.org) to develop DEG‐encoded proteins and PPI networks. Thereafter, Cytoscape software was utilized to construct protein interaction relationship networks and analyze the interaction relationship of the candidate DEGs encoding proteins. Finally, the Network Analyzer plug‐in was used to calculate node degree (the numbers of interconnections) to filter hub genes of PPI, with the corresponding proteins in the central nodes being the potential core proteins and key candidate genes that could have significant physiological regulatory functions.

### 2.12 Validation of sequence data

GAPDH and IGFBP2 mRNA expression levels were measured using real‐time quantitative PCR (RT–PCR) and analyzed using the 2^−ΔΔCT^ method. RNA from the mouse hippocampus was extracted using the RNAiso Plus kit (TaKaRa) according to the manufacturer's instructions. Isolated RNA molecules were transcribed into cDNA using the PrimeScript RT reagent Kit with gDNA Eraser (TaKaRa), and this cDNA was used as a template in further PCR analyses. GAPDH levels were used as the internal control. Quantitative real‐time PCR analysis was performed using a CFX96 Real‐Time PCR Detection System (Bio‐Rad) with a TB Green® Premix Ex Taq™ II (Tli RNaseH Plus) Kit (TaKaRa) following the manufacturer's instructions. All qRT‐PCRs were performed in triplicate, and the relative expression values were normalized to the internal control. The primers were designed according to the NCBI database: GAPDH (F: CGTCCCGTAGACAAAATGGT; R: GAATTTGCCGTGAGTGGAGT); IGFBP2 (F: CAAGCATGGCCGGTACAA; R: CGGTATTGGGGTTCACACAC).

### 2.13 Statistical analysis

All data were analyzed using GraphPad Prism (GraphPad Software, www.graphpad.com). All data were first tested for normality by the Kolmogorov–Smirnov test. The unpaired *t* test (two‐tailed) was used to analyze the data that passed the normality test, including the results of Western blotting, Y‐maze, self‐grooming bouts, and induced self‐grooming test. The Mann–Whitney test was applied for nonnormally distributed data of time spent in self‐grooming, reversal T‐maze, and elevated plus maze. Two‐way repeated‐measures ANOVA was used to analyze time spent in different chambers and sniffing time in the social approach test and marble burying test. All data points represent different samples. All data are presented as the mean ± standard error of the mean (SEM). For all data, the statistically significant *p* values are shown as *p < 0.05, **p < 0.01, ***p < 0.001, and ****p < 0.0001.

## 3 RESULTS

### 3.1 Generation and characterization of *Nestin‐Pax2* mice

First, we generated Nestin‐CreER: *Pax2* ^flox/+^ mice by crossing Nestin‐CreER mice with *Pax2* ^flox/flox^ (*Pax2* flox) mice. Nestin‐CreER: *Pax2* ^flox/+^ mice were further crossed with *Pax2* ^flox/flox^ mice to obtain Nestin‐CreER: *Pax2* ^flox/flox^ (*Nestin Pax2*) mice (Figure 1B). The length of the Cre site in *Nestin‐Pax2* mice was 272 bp, while that in *Pax2* flox mice was not, as shown in Figure 1C. The knockout of *Pax2* resulted in the loss of *Pax2* protein expression. Decreased *Pax2* expression in *Nestin‐Pax2* mice was supported by Western blotting (1 ± 0.09 vs 0.43 ± 0.06, *t* = 5.3, *p* < 0.01, Figure 1D, Figure S1). Thus, we successfully constructed a *Pax2* nervous system‐specific knockout mouse model.

Both *Nestin‐Pax2* mice and *Pax2* flox mice share a number of key features, including general health and empty cage behaviors (Table S1). No significant differences were observed in body weight between these two groups (17 ± 0.54 vs. 15 ± 0.84, *t* = 1.7, *p* = 0.1085). The ear‐twitch reflex, eye‐blink reflex, postural reflex, righting reflex, and whisker‐touch reflex indicated normal neurological reflexes in all mice. Likewise, normal visual and auditory functions were seen in all mice.

### 3.2 Increased repetitive self‐grooming and impaired reversal learning in *Nesttin‐Pax2* mice

**FIGURE 2:**
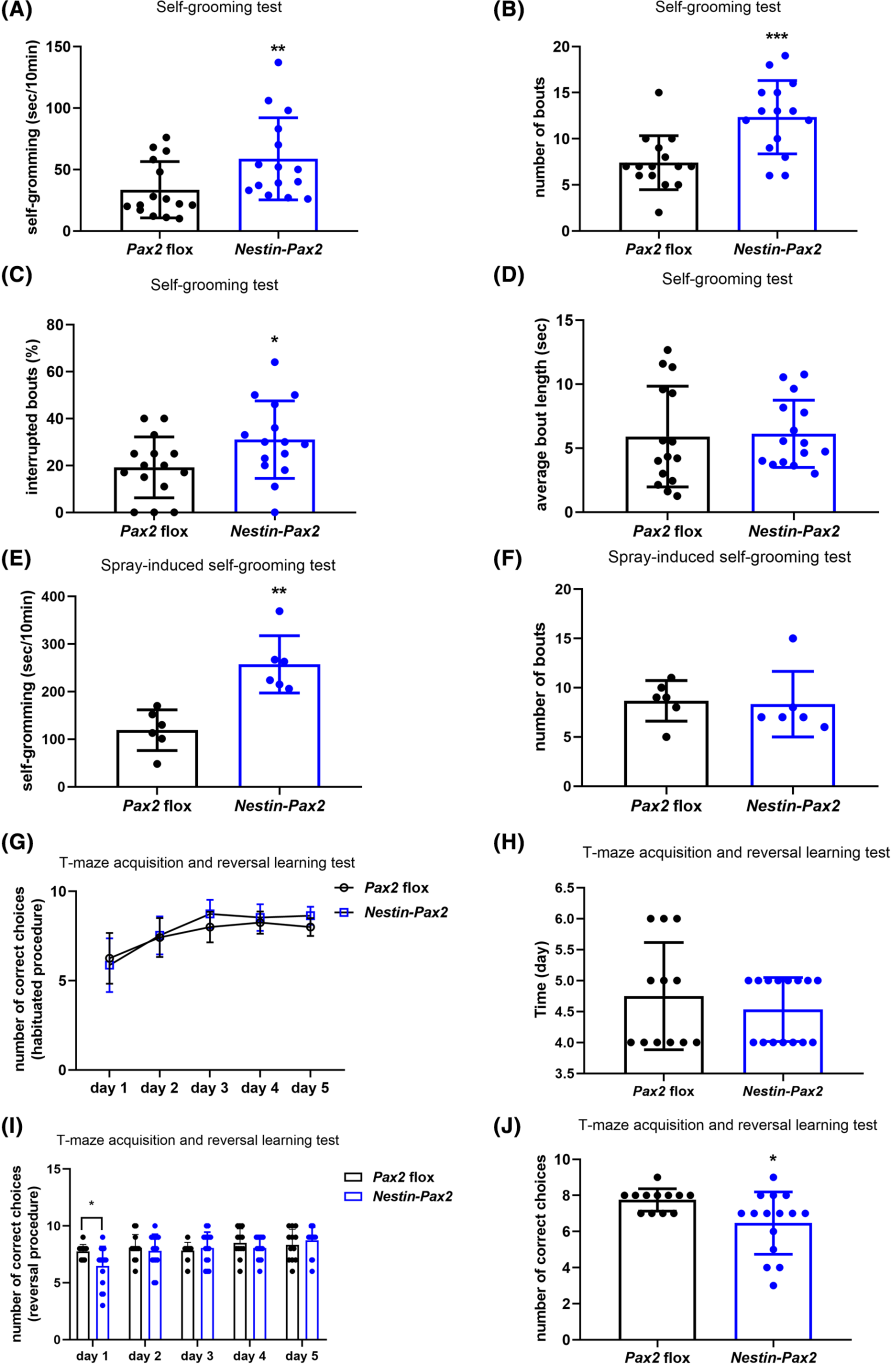
*Nestin‐Pax2* mice showed increased self‐grooming and impaired reversal learning. (A) *Nestin‐Pax2* mice spent more time self‐grooming than *Pax2* flox mice. (B) The number of bouts increased in *Nestin‐Pax2* mice compared to *Pax2* flox mice. (C) The proportion of interrupted bouts was higher in *Nestin‐Pax2* mice than in *Pax2* flox mice. (D) There was no difference in average bout length between *Pax2* flox mice and *Nestin‐Pax2* mice. *n* = 15 *Pax2* flox mice, including eight males and seven females; *n* = 16 *Nestin‐Pax2* mice, including six males and nine females. In the spray‐induced self‐grooming test, *Nestin‐Pax2* mice spent more time self‐grooming (E) but showed a normal number of bouts (F) compared with *Pax2* flox mice. *n* = 6 *Pax2* flox mice, including three males and three females; *n* = 6 *Nestin‐Pax2* mice, including two males and four females. (G) On Day 1–Day 6 of the T‐maze, *Pax2* flox mice and *Nestin‐Pax2* mice both display normal acquisition learning of the reward, as indicated by the number of correct choices. (H) The number of days needed to reach the criteria for reversal learning was not significantly different between *Pax2* flox mice and *Nestin‐Pax2* mice. (I) Total number of correct choices during the 5‐day reversal learning session. (J) On reversal Day 1, *Nestin‐Pax2* mice made fewer correct choices than *Pax2* flox mice. *n* = 12 *Pax2* flox mice, including eight males and four females; *n* = 15 *Nestin‐Pax2* mice, including nine males and six females. Data are shown as the mean ± SEM. *p < 0.05, **p < 0.01, ***p < 0.001.

We next examined behavioral phenotypes in *Nestin‐Pax2* mice. These mice displayed significantly increased self‐grooming in the test, as shown by the time spent in self‐grooming (34 ± 5.9 vs 59 ± 8.6, U = 51, *p* < 0.01, Figure 2A) and self‐grooming bouts (7.4 ± 0.75 vs 12 ± 1.0, *t* = 3.9, *p* < 0.001, Figure 2B). Following the additional analysis of self‐grooming, a significant increase in the proportion of interrupted bouts in *Nestin‐Pax2* mice was recorded (19 ± 3.4 vs 31 ± 4.3, *t* = 2.2, *p* < 0.05, Figure 2C). However, no significant difference in average bout length (5.9 ± 1.0 vs 6.1 ± 0.68, U = 101, *p* > 0.05, Figure 2D) between the two groups was evident. In the spray‐induced self‐grooming test, *Nestin–Pax2* mice spent more time in self‐grooming than *Pax2* flox mice (119 ± 18 vs 257 ± 25, *t* = 4.6, *p* < 0.01, Figure 2E). However, there was no difference in self‐grooming bouts (8.7 ± 0.84 vs 8.3 ± 1.4, *t* = 0.21, *p* = 0.8390, Figure 2F).

In the following test, we used performance in the reversed T‐maze to determine the higher‐order repetitive behaviors of *Nestin‐Pax2* mice. As shown in Figure 2G, all mice reached the criterion on Day 6 and were moved into the training session of the reversal learning test. There was no significant difference in the number of days to reach the 80% right position criterion between *Pax2* flox mice and *Nestin‐Pax2* mice (4.8 and 4.5 days, repetitively) (4.8 ± 0.25 vs 4.5 ± 0.13, U = 81, *p* > 0.05, Figure 2H). In the reversal procedure of the T‐maze, *Nestin‐Pax2* mice made a greater number of errors on the first day of the reversal test than *Pax2* flox mice (7.8 ± 0.18 vs 6.5 ± 0.45, U = 46, *p* < 0.05, Figure 2J). Figure 2I shows the number of errors on each day throughout the 5‐day reversal learning test.

### 3.3 Normal in social approach, marble burying, Y‐maze, and elevated plus maze in *Nestin‐Pax2* mice

**FIGURE 3:**
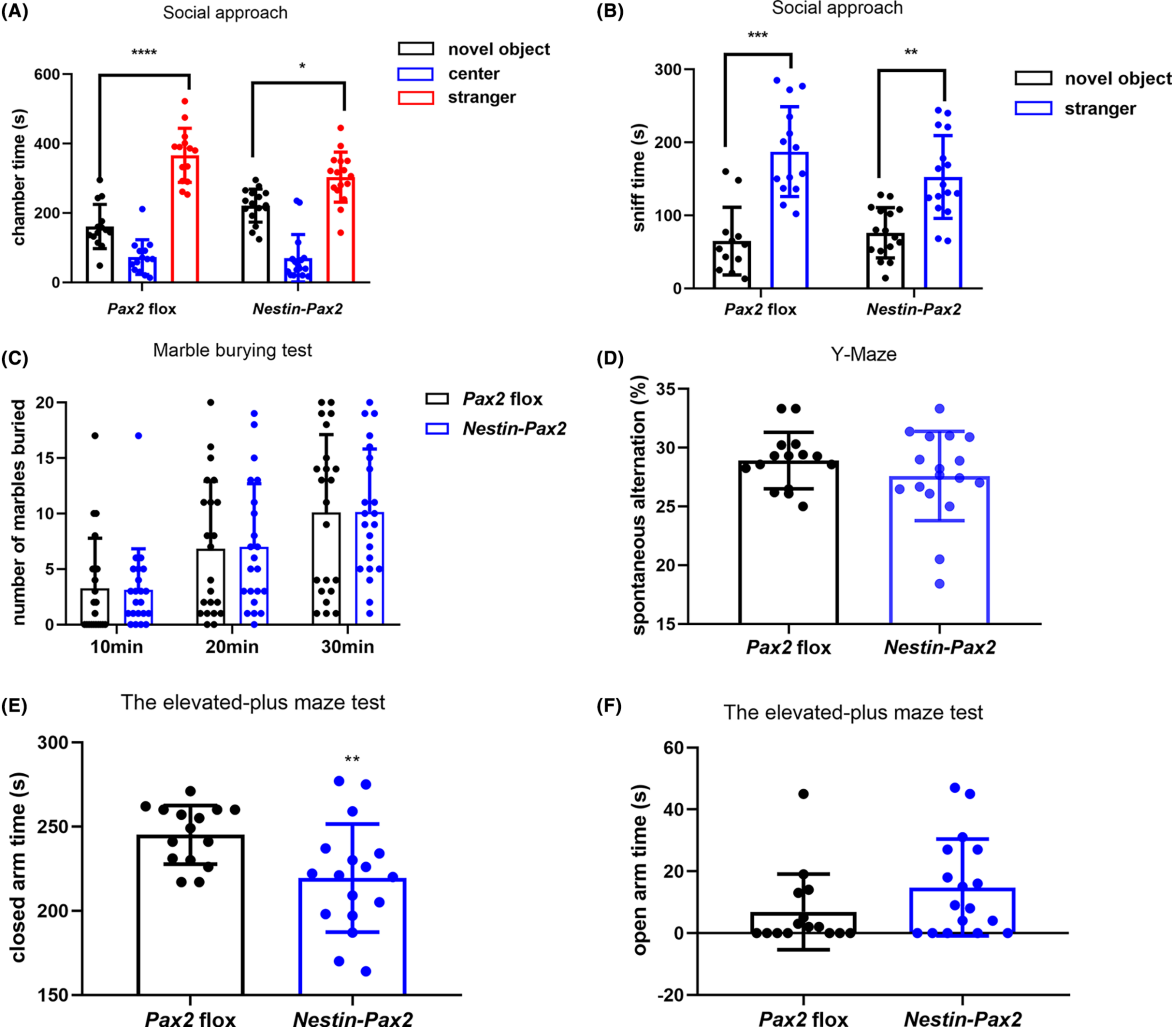
*Nestin‐Pax2* mice showed normal social approach, marble burying, Y‐maze, and elevated plus maze performance. There was no difference in the time spent staying in the chamber of the stranger mouse (A) or sniffing the stranger mouse (B) between *Pax2* flox mice and *Nestin‐Pax2* mice. *n* = 14 *Pax2* flox mice, including seven males and seven females; *n* = 16 *Nestin‐Pax2* mice, including six males and 10 females. The result (C) showed that there was no difference in the number of marbles buried between *Pax2* flox mice and *Nestin‐Pax2* mice. *n* = 22 *Pax2* flox mice, including 10 males and 12 females; *n* = 22 *Nestin‐Pax2* mice, including 11 males and 11 females. There was no genotype difference detected in the spontaneous alternations between *Pax2* flox mice and *Nestin‐Pax2* mice (D). *n* = 15 *Pax2* flox mice, including eight males and seven females; *n* = 17 *Nestin‐Pax2* mice, including seven males and 10 females. Compared with *Pax2* flox mice, *Nestin‐Pax2* mice spent less time in the closed arm in the elevated plus‐maze (E), and there was an increasing trend in the time spent in the open arm (F). *n* = 15 *Pax2* flox mice, including eight males and seven females; *n* = 17 *Nestin‐Pax2* mice, including seven males and 10 females. Data are shown as the mean ± SEM. *p < 0.05, **p < 0.01, ***p < 0.001, ****p < 0.0001.

In the social approach test, there was a significant difference in chamber time (*F*_2,30_ = 116, *p* < 0.001) but not between *Pax2* flox and *Nestin‐Pax2* mice (*F*_1,15_ = 0.025, *p* = 0.8763) (Figure 2A). Similarly, there was a positive influence of sniffing time (*F*_1,15_ = 58, *p* < 0.0001) but not of genotype (*F*_1,15_ = 0.77, *p* = 0.3936) (Figure 2B). In another test for lower‐order repetitive behavior named the marble burying test, *Nestin‐Pax2* mice displayed normal levels for marbles buried (Figure 3C).

For the experimental evidence of learning and memory ability, the spontaneous alternation in the Y‐maze of these two types of mice showed no significant difference (29 ± 0.62 vs 28 ± 0.92, *t* = 1.2, *p* = 0.2554, Figure 3D). In the elevated plus maze test, *Nestin‐Pax2* mice spent less time in the closed arm (245 ± 4.5 vs 219 ± 7.8, *t* = 2.8, *p* < 0.01, Figure 3E) and more time in the open arm (6.9 ± 3.2 vs 15 ± 3.8, U = 85, *p* = 0.1015, Figure 3F).

### 3.4 Increased number of projection fibers in the mPFC projecting to the CA1/Sub/BLA

**FIGURE 4:**
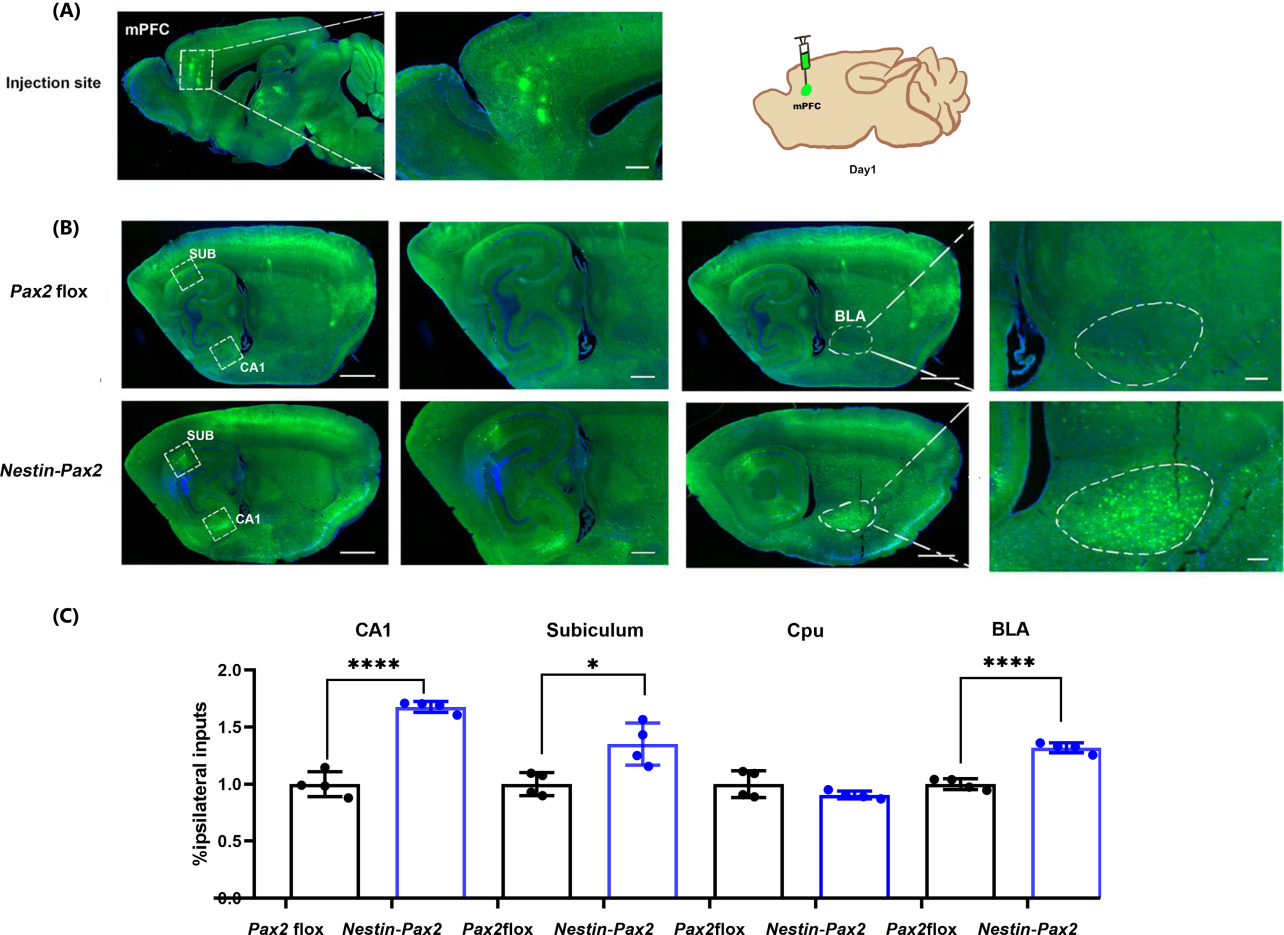
Nerve fiber connectivity of the mPFC→CA1/Sub/BLA neural circuitry. (A) Schematic diagram and typical images of injection sites and viral expression within the mPFC of *Pax2* flox mice. [Scale bars: 1 mm (left), 500 μm (right).] (B) The first column shows HSV‐EGFP‐labeled projection fibers from the mPFC‐tracked CA1 and subiculum in *Pax2* flox and *Nestin‐Pax2* mice. The second column is the enlargement of the rectangular frame on the left. [Scale bars: 1 mm (left), 500 μm (right).] The third column shows HSV‐EGFP‐labeled projection fibers from the mPFC tracked to the BLA in *Pax2* flox and *Nestin‐Pax2* mice. The fourth column is the enlargement of the left area. [Scale bars: 1 mm (left), 200 μm (right).] (C) *Nestin‐Pax2* mice have increased numbers of projection fibers in CA1, subiculum and BLA compared to *Pax2* flox mice. *n* = 4 *Pax2* flox mice, including one male and three females; *n* = 4 *Nestin‐Pax2* mice, including one male and three females. Data are shown as the mean ± SEM. *p < 0.05, ****p < 0.0001.

HSV‐EGFP was injected into the mPFC of *Pax2* flox and *Nestin‐Pax2* mice (Figure 4A). Compared to *Pax2* flox mice, we found a large number of EGFP‐labeled projection fibers in the CA1 and subiculum in *Nestin‐Pax2* mice. Further tracer virus tracing indicated that the number of fibers transmitted between the mPFC and BLA was significantly increased in *Nestin‐Pax2* mice (CA1: *t* = 11, *p* < 0.0001; subiculum: *t* = 3.4, *p* = 0.0153; BLA: *t* = 9.8, *p* < 0.0001; Figure 4B,C). Meanwhile, we observed that projection fibers were present in the striatum of both the *Pax2* flox and *Nestin‐Pax2* mice, with no statistically significant differences (*t* = 1.5, *p* = 0.1733; Figure 4C, Figure S2).

### 3.5 Decreased IGFBP2 in the hippocampus of *Nestin‐Pax2* mice

**FIGURE 5:**
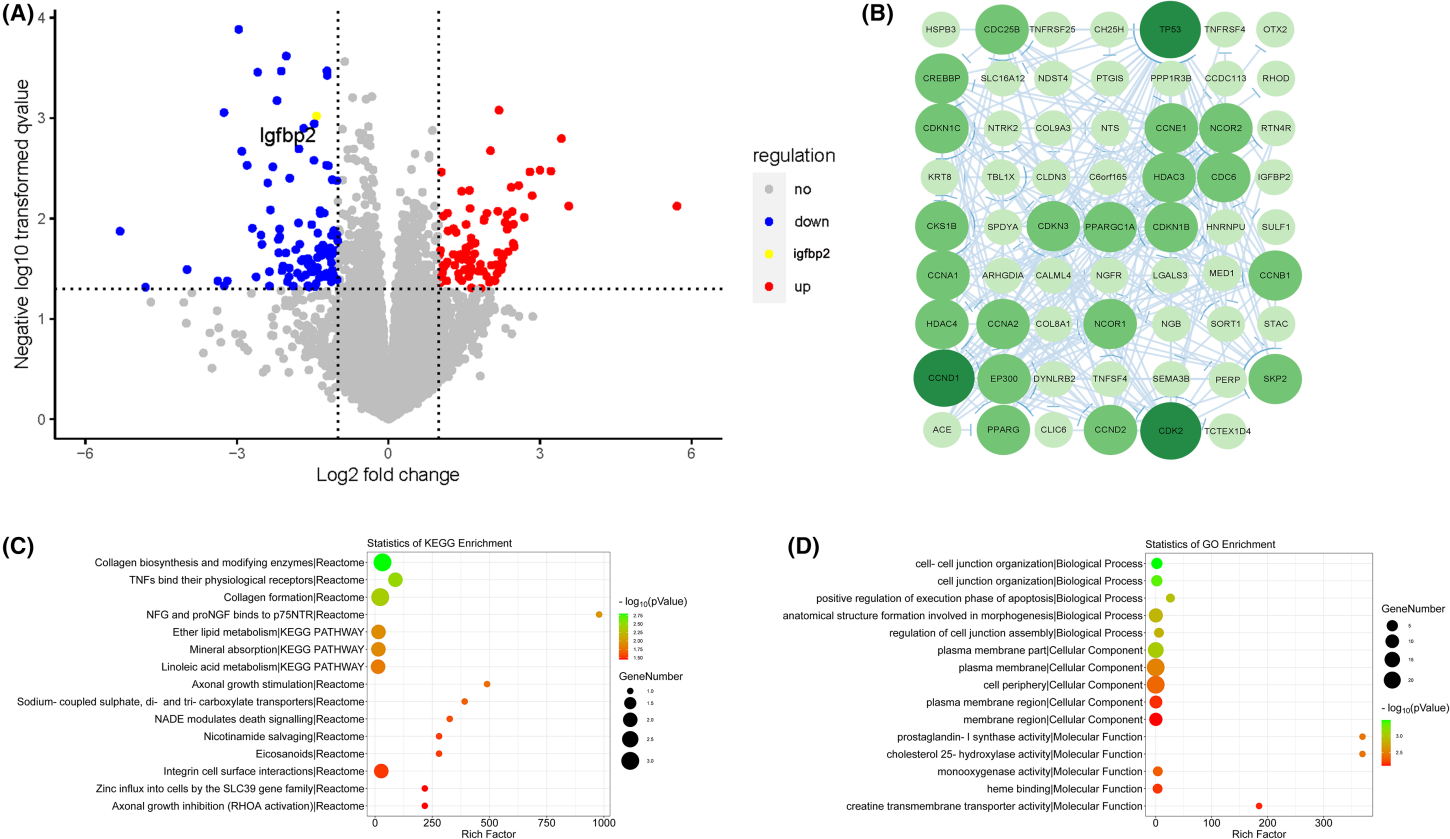
DEGs for volcano mapping, scoring of KEGG richness and GO enrichment analysis and protein interaction clusters in DEGs. (A) Volcano plot of DEGs. The horizontal axis shows the log2 value of the expression difference multiple, and the vertical axis shows the *p* value after taking the negative logarithm in differential expression analysis. Red dots represent upregulated differentially expressed genes, green dots represent downregulated differentially expressed genes, black dots represent genes with no significant differences, and yellow dot represents IGFBP2. (B) Protein interaction community of DEGs. The node is the gene where the protein is located, the larger the node represents the more nodes connected to it, the circle is the node, and the size represents the size of the degree. (C) KEGG enrichment score of DEGs. The horizontal coordinate indicates the result of the rich factor in the enrichment result; the larger the value is, the greater the enrichment. The color represents the *p* value; the smaller the value is, the more reliable the enrichment. The size of the circle represents the input number in the enrichment result; the larger the input number is, the larger the circle. (D) GO enrichment analysis of DEGs. The horizontal coordinate indicates the rich factor result in the enrichment results, and a larger value indicates greater enrichment. The color represents the *p* value; the smaller the value is, the more reliable the enrichment. The size of the circle represents the input number of the enrichment result; the larger the input number is, the larger the circle.^21^

In addition, to elucidate the influence of *Pax2* at the molecular level in mice, we used transcriptome sequencing to show the differential expression of *Pax2* in mice at the transcriptome level. The results confirmed that the expression of 85 genes was significantly upregulated and that the expression of 96 genes was significantly downregulated in the experimental *Nestin‐Pax2* mice compared to the control *Pax2* flox mice (Figure 5A). Interestingly, there were also differences in neurotrophic factors, such as insulin‐like growth factor binding protein‐2 (IGFBP2), between *Pax2* flox mice and *Nestin‐Pax2* mice (1.0 ± 0.06 vs 0.54 ± 0.09, *t* = 4.453, *p* < 0.05, Figure S3), which play a vital role in neural growth and synapse formation.

The results of the GO assessment confirmed that the DEGs were commonly involved in apoptosis, microtubule‐associated complexes, and oxidoreductase regulatory signaling pathways (Figure 5D). The results of GO assessment confirmed that the differentially expressed genes were typically involved in apoptosis, microtubule‐associated complexes, and regulatory signaling pathways of oxidoreductase activity (Figure 5D).

The results of the KEGG assessment confirmed that the differentially expressed genes were broadly involved in signaling pathways such as axonal overgrowth stimulation, axonal overgrowth inhibition (RHOA activation), p75NTR‐recruiting signaling complexes, and NADE modulation of death signaling (Figure 5C). To determine more about the hub genes in the protein–protein interaction community encoded using DEGs, the STRING database and the Cytoscape software program were used to assess the PPI community. Each node represents a protein. The greener the hue of the gene, the higher the core note and vice versa. The results confirmed that the 20 hub genes screened were as follows: TP53, CDK2, CCND1, CDC25B, CREBBP, CDKN1C, CCNE1, NCOR2, HDAC3, CDC6, CKS1B, CDKN3, PPARGC1A, CDKN1B, CCNA1, CCNB1, HDAC4, CCNA2, NCOR1, CCND1, EP300, SKP2, PPARG, and CCND2 (Figure 5B).

## 4 DISCUSSION

While the genetic heterogeneity and intricacy of RRBs has complicated the question of pathophysiological mechanisms, abundant evidence suggests that transcription factors play an important role in the process of neurodevelopmental disorders.^17^, ^18^ *Pax2* is one of the transcription factors expressed in many tissues,^8^, ^19^ and mutations in *Pax2* lead to many diseases.^9^, ^10^, ^11^, ^20^ In previous studies, we found increased self‐grooming behavior and impaired spatial and memory abilities in *Pax2* ^+/−^ mice.^12^, ^13^ In the present study, to investigate whether and how *Pax2*‐specific knockout in the nervous system affects the phenotypic behavior of mice, we assessed the behavioral phenotype, neural circuitry tracing, and RNA‐seq in the hippocampus of *Nestin‐Pax2* mice and *Pax2* flox mice. The results show increased RRBs in *Nestin‐Pax2* mice, alterations in the mPFC**→**CA1/BLA neural circuitry, and deficits of IGFBP2 in the hippocampus.

Here, we identified the behavioral phenotypes of *Nestin‐Pax2* mice. We observed increased RRBs in both lower‐order and higher‐order. *Nestin‐Pax2* mice showed increased self‐grooming in the empty cage but normal social approach. These results are similar to those of *Pax2* ^+/−^ mice and are also consistent with those of *Pax2* mutation patients with ASD.^20^ In the spray‐induced self‐grooming test, *Nestin‐Pax2* mice also showed increased self‐grooming. In another test for lower‐order RRBs called the marble burying test, the two groups of mice showed the same results. This is probably due to the long‐term repetitive self‐grooming movement in the cage, thus ignoring the marbles. The impaired reversal learning in *Nestin‐Pax2* mice suggests that there is a resistance to change in routine, which implies a stereotype in perception. Overall, these results indicate that knockout of *Pax2* in the nervous system leads to strong RRBs in both lower‐order and higher‐order during different tests. In the elevated plus maze test, *Nestin‐Pax2* mice spent more time in the open arm, which indicated that *Nestin‐Pax2* mice showed hyperactivity and hyperexploration. One previous study showed that self‐grooming can alleviate the stress‐induced anxious state.^21^ Therefore, it is reasonable to speculate that increased self‐grooming will result in less anxiety in *Nestin‐Pax2* mice. Increased RRBs have been observed in many patients and animal models of neurodevelopmental and neurodegenerative disorders,^1^, ^4^, ^22^, ^23^ but the exact mechanisms of RRBs are still unclear.

Previous research has shown the role of the cortico‐basal ganglia circuitry in the etiology of motor dysfunction in neuropsychiatric disorders, such as ASD and OCD, mainly RRBs. The generation of symptoms in RRBs may require different neural circuitry.^24^ An influential hypothesis in this area describes an imbalance between excitatory and inhibitory neuronal control leading to increased RRBs.^15^ A more recent hypothesis suggests that RRBs may be explained by a generalized dysfunction of the dopamine diffusion systems.^25^ Therefore, research over the last decade has further emphasized the existence of multiple causes for the pathophysiological mechanisms by which RRBs arise. The advantage of examining the neural circuitry mechanism of a disorder is that the convergent pathological consequences for behavior can be revealed. In this study, our results showed that an increase in the number of projection fibers in the mPFC→CA1/BLA neural circuitry is involved in the growth of RRBs in *Nestin‐Pax2* mice. Since the CSTC neural circuitry in RRBs has been extensively studied and there is extensive connectivity between the mPFC and other brain regions,^26^, ^27^ we used the mPFC as an injection site to search for differences in neural circuitry. Numerous studies have shown that there are strong multisynaptic connections between the mPFC and the hippocampus.^28^ Additionally, in the RRBs, neurons in the BLA and hippocampus are active,^16^ indicating their neural correlation. The mPFC forms nerve fiber connectivity with both the hippocampus and the BLA. It is bidirectionally connected to the amygdala^29^ and simultaneously receives projections from the hippocampus.^30^ Some studies have shown that the functional connectivity of the BLA and mPFC was significantly increased in obsessive‐compulsive checking behavior, suggesting that the mPFC→BLA pathway plays an important role in obsessive‐compulsive checking behavior.^31^

To further investigate the neurobiological mechanism of *Pax2* gene knockout in the nervous system leading to increased RRBs and impaired neural circuitry of the hippocampus, we used RNA‐seq technology to observe the DEGs and signaling pathways. RNA‐seq revealed a total of 181 DEGs between *Pax2* flox mice and *Nestin‐Pax2* mice. Many of them are related to neurotrophic factors, including insulin‐like growth factor binding protein‐2 (IGFBP2). IGFBP2 is one of the IGFBPs that can form a complex with insulin‐like growth factor (IGF) in the circulation to modulate IGF levels.^32^, ^33^, ^34^, ^35^, ^36^ Previous studies also reported that the expression of IGFBP2 and IGF was coordinated in the cerebellum but not in the hippocampus,^37^, ^38^ suggesting that IGFBP2 plays an important role in the hippocampus independent of IGF binding. IGFBP2 is most abundant in cerebrospinal fluid (CSF) and is highly expressed in pyramidal neurons and GABAergic interneurons in the developing hippocampus.^39^ There is also a significant difference in CSF IGFBP2 levels in Alzheimer's disease, which shows cognitive dysfunction.^40^ Patients with autism, a disorder with RRBs and impaired socialization, have also been shown to have an abnormal IGFBP system.^32^, ^41^ In our studies, we observed decreased IGFBP2 levels in the hippocampus of *Nestin‐Pax2* mice. In another study, S. Khan et al. (2019)^42^ showed that knockout of IGFBP2 can affect spinal growth and neuronal proliferation; meanwhile, weaker LTP and disruption of excitation–inhibition balance in the hippocampus were also observed in IGFBP2^−/−^ mice, resulting in impaired cognitive behavior. We speculated that knockout of *Pax2* in the nervous system affects the expression of IGFBP2 in the hippocampus, thereby disrupting the excitation‐inhibition balance by affecting synaptic plasticity and neural circuitry in the hippocampus, resulting in RRBs.

## 5 CONCLUSION

In conclusion, the construction of *Pax2* nervous system‐specific knockout mice allowed us to further investigate the role of *Pax2* in RRBs. *Nestin‐Pax2* mice showed increased RRBs in both lower‐order and higher‐order, alterations in the mPFC**→**CA1/BLA neural circuitry and a reduction in IGFBP2 in the hippocampus. However, how the *Pax2* gene participates in IGFBP2 signaling and regulates neural circuitry in the hippocampus remains unclear. Exploration of potential neural circuitry and the relationship between IGFBP2 and *Pax2* may facilitate a better understanding of RRBs and provide new mechanistic insights for potential therapeutic interventions.

## CONFLICT OF INTEREST STATEMENT

The authors declare that they have no conflict of interest.

## Supporting information


Data S1.

## Data Availability

The data that support the findings of this study are available from the corresponding author upon reasonable request.
