# Supplementary material for: Impaired neural circuitry of hippocampus in Pax2 nervous system‐specific knockout mice leads to restricted repetitive behaviors
Source: CNS Neurosci Ther. 2023 Oct 3;30(4):e14482. doi: 10.1111/cns.14482 (PMC11017408; doi:10.1111/cns.14482)
Supplement: Supplementary file 1 — Data S1. [file CNS-30-e14482-s001.pdf]

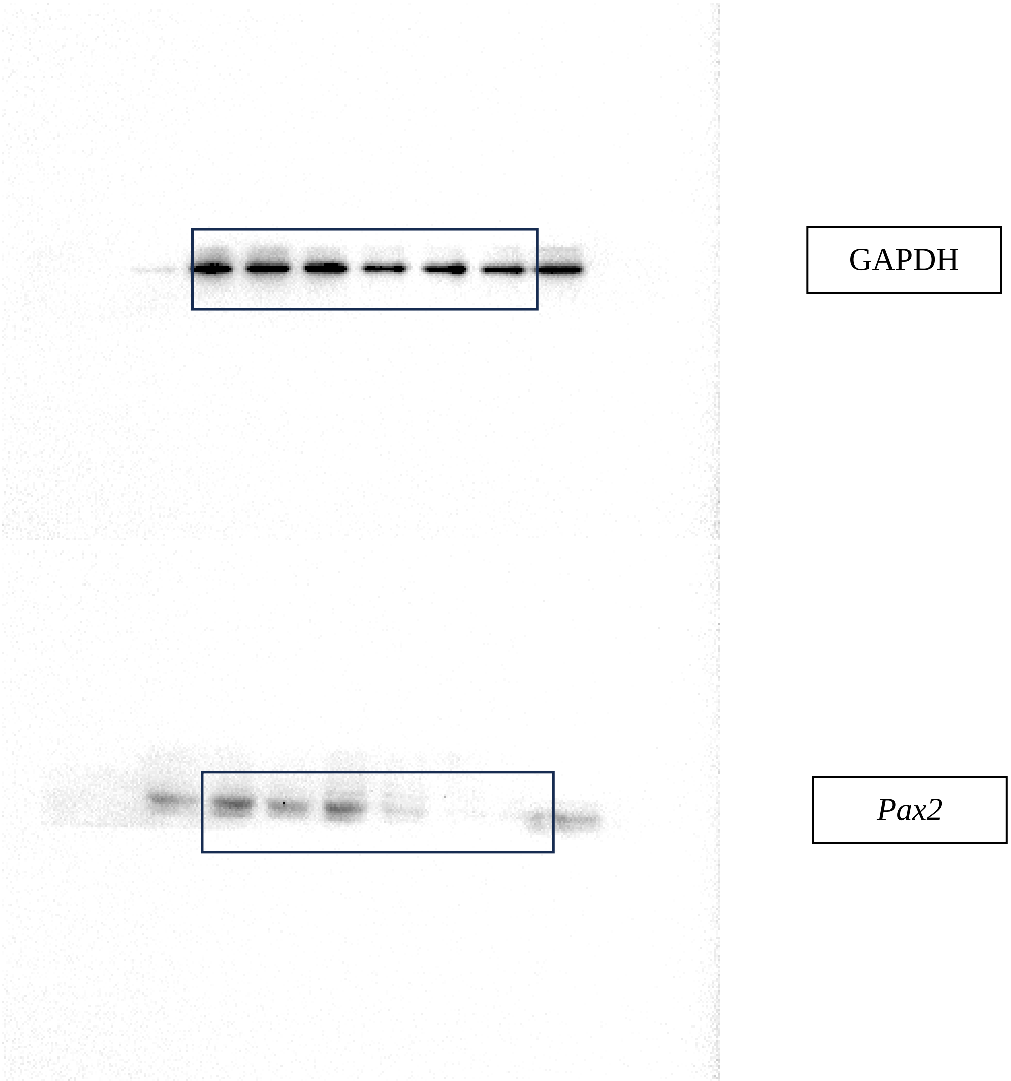

Supplementary Figure 1: Full unedited gel/blot for Figure 1D. The lanes in the black frame appear in the cropped image in the manuscript.

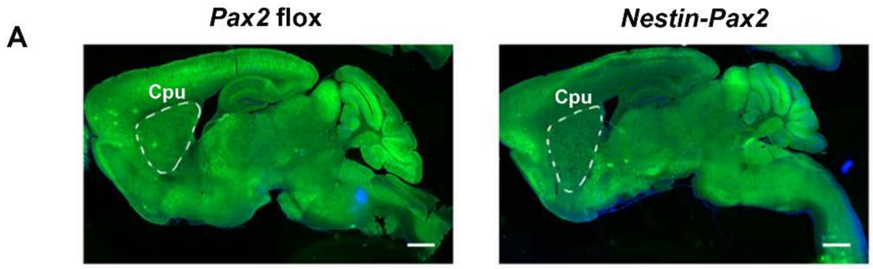

Supplementary Figure 2: Nerve fiber connectivity of the mPFC→Cpu neural circuitry. EGFP-labeled projection fibers within the striatum traced from the mPFC of *Pax2* flox mice and *Nestin-Pax2* mice. n=4 *Pax2* flox mice, including 1 male and 3 females; n=4 *Nestin-Pax2* mice, including 1 male and 3 females. [Scale bars: 1 mm.] Data are shown as mean ± SEM. ****$P < 0.0001$.

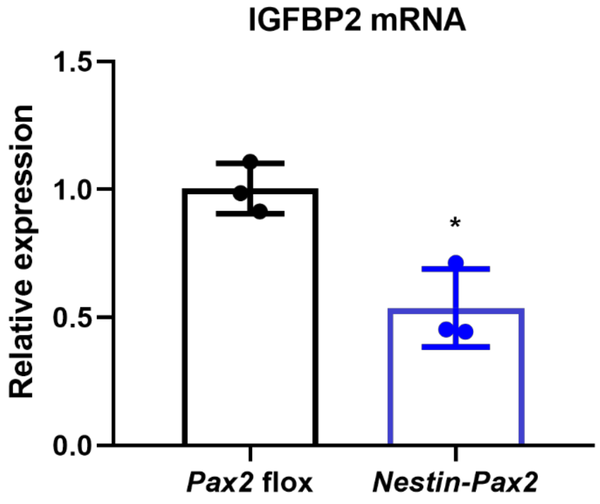

Supplementary Figure 3: Sequencing data validation. The results showed the mRNA level of IGFBP2 in the hippocampus of *Nestin-Pax2* mice was significantly lower than that of the control group. n=3 *Pax2* flox mice; n=3 *Nestin-Pax2* mice. Data are shown as mean ± SEM. *$P < 0.05$.

Supplementary Table 1: General health check and neurological screening

Genotypes	*Pax2* flox (N=10)	*Nestin-Pax2* (N=10)	*P*-value
General health			
body weight	17±0.54	15±0.84	NS
Fur and whisker Condition (3-point scale)	3	3	NS
limb and body tone (3-point scale)	2	2	NS
Sensory function			
visual placing test (%)	100	100	NS
preyer reflex test (%)	100	100	NS
neurological screening test			
ear-twitch reflex (%)	100	100	NS
eye-blink reflex (%)	100	100	NS
postural reflex (%)	100	100	NS
righting reflex (%)	100	100	NS
whisker-touch reflex (%)	100	100	NS

Data are expressed as percentages, 3-point rating scales, or mean±SEM. NS, not significant.
